# Supplementary material for: Affective Enhancement of Episodic Memory Is Associated With Widespread Patterns of Intrinsic Functional Connectivity in the Brain Across the Adult Lifespan
Source: Front Behav Neurosci. 2022 Jun 27;16:910180. doi: 10.3389/fnbeh.2022.910180 (PMC9271876; doi:10.3389/fnbeh.2022.910180)
Supplement: Supplementary file 1 [file Data_Sheet_1.pdf]

Katsumi & Moore: Supplementary Materials

Supplementary Materials and Methods

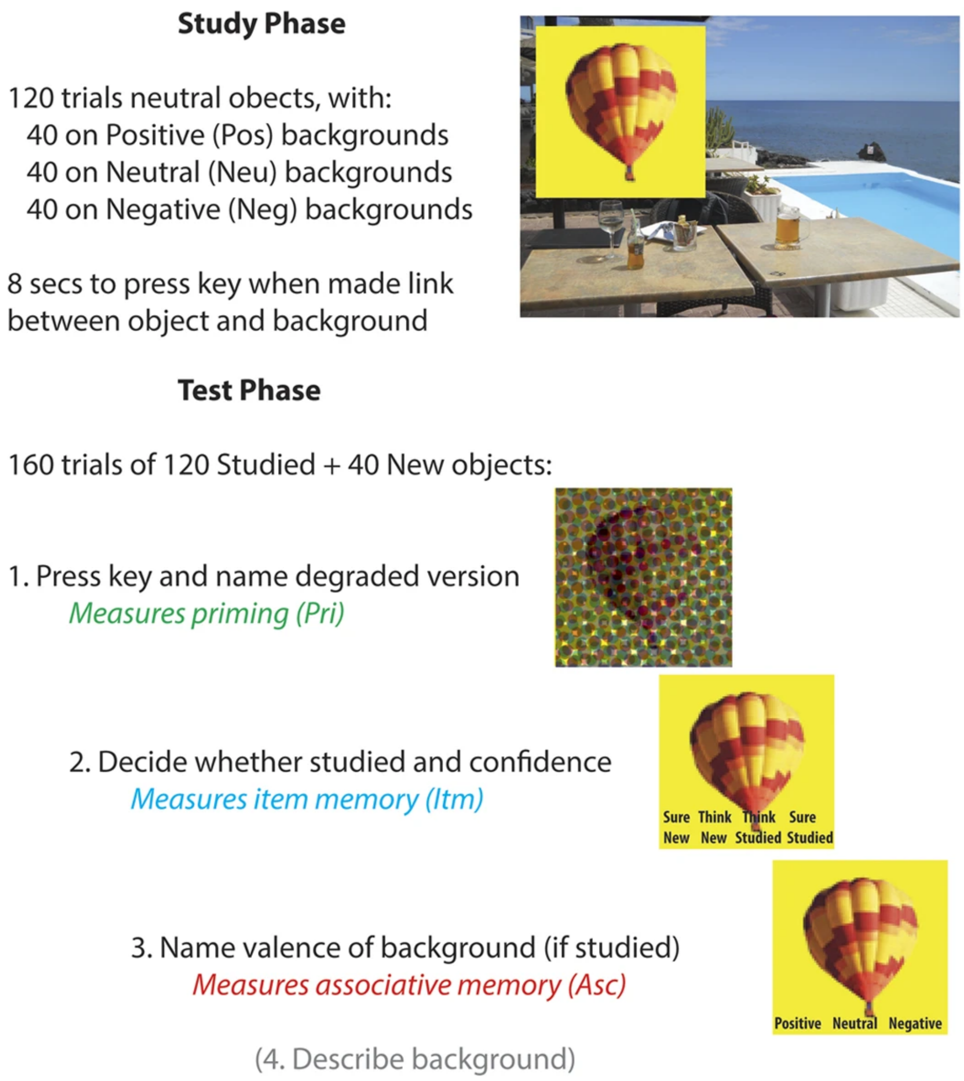

Supplementary Figure 1. Summary of Study and Test phases. Figure reproduced from Henson et al. (2016), with permission.

Justification of the number of components in functional connectivity MVPA

Functional connectivity MVPA requires the user to specify the number of principal components (N_c) to use in characterizing between-participants variability in whole-brain voxel-to-voxel connectivity patterns. Convention in the literature is to define N_c based on the participant-to-component ratio (Arnold Anteraper et al., 2019, 2020; Guell et al., 2020; Katsumi et al., 2021; Kawagoe et al., 2019; Takamiya et al., 2021; Whitfield-Gabrieli et al., 2016). In the current study, we initially performed first-level MVPA using 15 components, approximately corresponding to a 20:1 ratio between the number of participants and that of components. In addition to the participant-to-component ratio, recent work has begun taking into account the individual and cumulative explained variance for each component (Morris et al., 2021). It is important to keep in mind that the inclusion of too many components might (1) make it difficult to detect an effect of a given size across the components in the omnibus F-test, and (2) begin to explain variance in white matter voxel connectivity that is considered noise in this work.

Supplementary Table 1 below shows the percentage explained variance in gray matter functional connectivity variability by each component as well as the cumulative percentage for each additional component. Beyond seven components, each additional component explains less than 1% of variance, with the total variance explained by the first seven components being ~92%. Based on these results, we reasoned that seven components would sufficiently represent the observed between-participants variability in whole-brain connectivity structure at each voxel.

Component	Variance explained (%)	Cumulative variance explained (%)
1	74.07	74.07
2	7.28	81.34
3	4.16	85.51
4	2.58	88.09
5	1.82	89.91
6	1.37	91.28
**7**	**1.04**	**92.32**
8	0.81	93.13
9	0.65	93.78
10	0.52	94.30
11	0.43	94.74
12	0.36	95.10
13	0.30	95.40
14	0.25	95.65
15	0.22	95.87

Supplementary Table 1. Individual and cumulative variance in gray matter connectivity variability explained by each MVPA component.

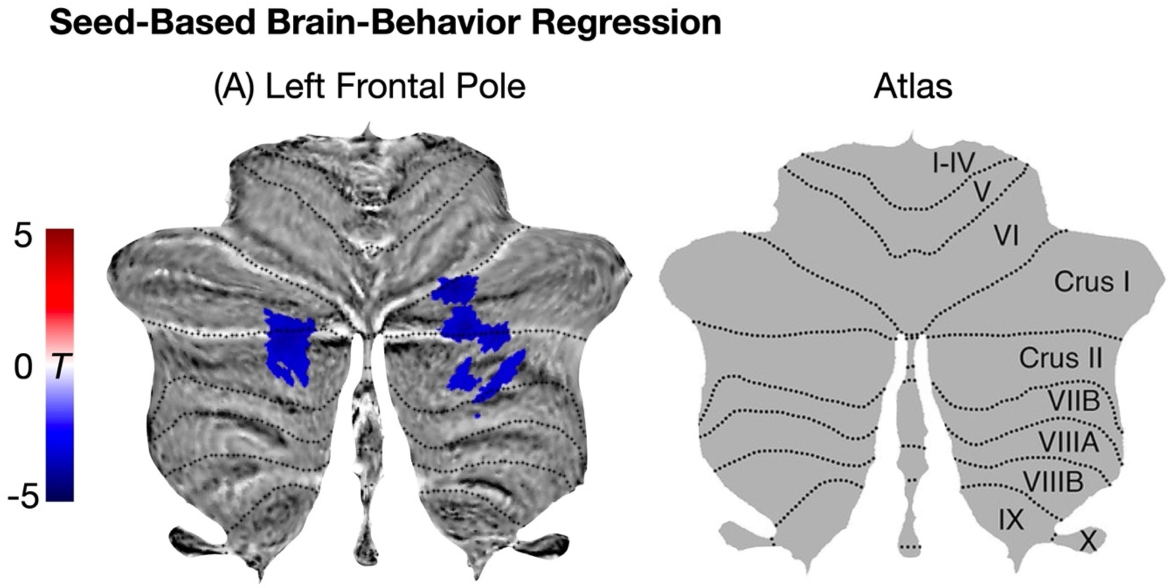

Supplementary Figure 2. Intrinsic functional connectivity correlates of item memory enhancement in the subcortex. Left panel: Clusters of suprathreshold voxels in the cerebellar cortex whose functional connectivity with the left frontal pole seed (see **Figure 2** in the main text) was negatively associated with item memory enhancement. These clusters are depicted on a flat map representation of the cerebellar cortex (Diedrichsen & Zotow, 2015). Right panel: Subregions of the cerebellar cortex depicted on a flat map. Reproduced from Guell et al. (2018), with permission.

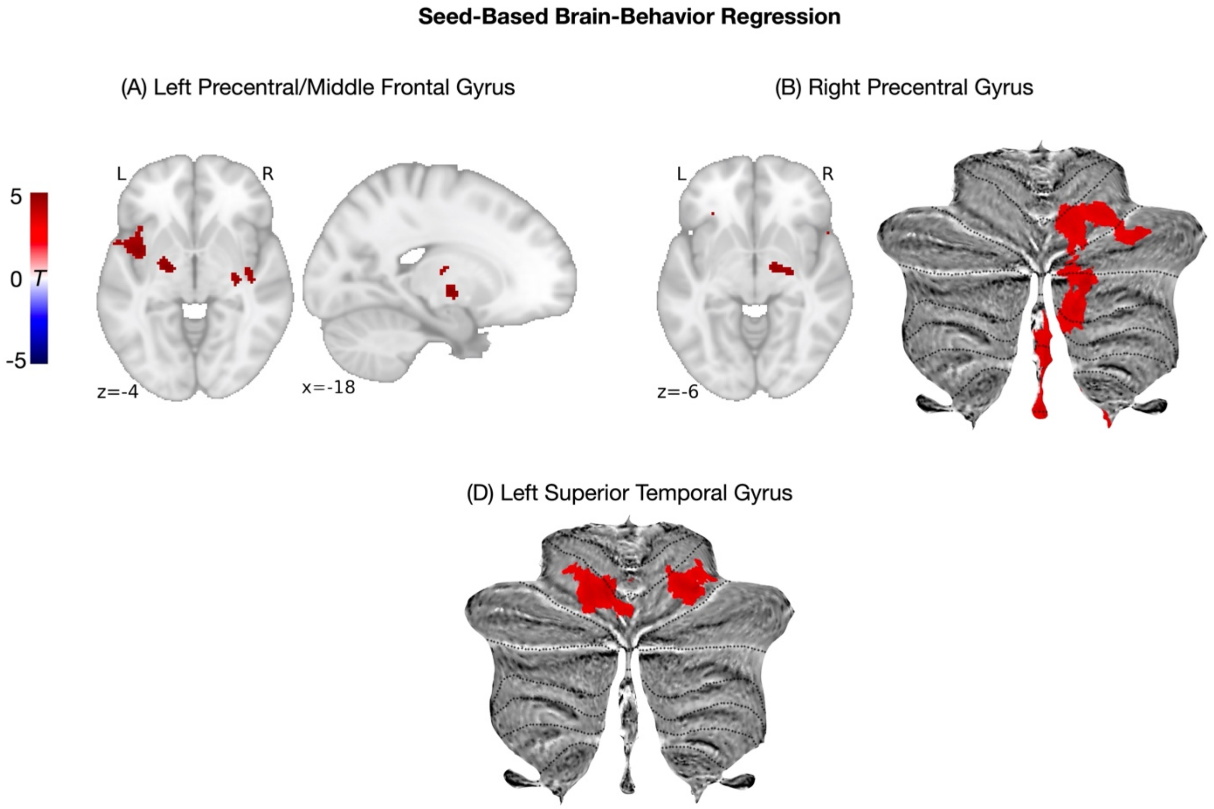

Supplementary Figure 3. Intrinsic functional connectivity correlates of associative memory enhancement in the subcortex. These volumetric and cerebellar flat map projections identify clusters of suprathreshold voxels whose functional connectivity with the seed regions (see **Figure 3** in the main text) was positively associated with associative memory enhancement.

References

Arnold Anteraper, S., Collin, G., Guell, X., Scheinert, T., Molokotos, E., Henriksen, M. T., Mesholam-Gately, R., Thermenos, H. W., Seidman, L. J., Keshavan, M. S., Gabrieli, J. D. E., & Whitfield-Gabrieli, S. (2020). Altered resting-state functional connectivity in young children at familial high risk for psychotic illness: A preliminary study. *Schizophrenia Research*, *216*, 496–503. https://doi.org/10.1016/j.schres.2019.09.006

Arnold Anteraper, S., Guell, X., D'Mello, A., Joshi, N., Whitfield-Gabrieli, S., & Joshi, G. (2019). Disrupted cerebrocerebellar intrinsic functional connectivity in young adults with high-functioning autism spectrum disorder: A data-driven, whole-brain, high-temporal resolution functional magnetic resonance imaging study. *Brain Connectivity*, *9*(1), 48–59. https://doi.org/10.1089/brain.2018.0581

Diedrichsen, J., & Zotow, E. (2015). Surface-based display of volume-averaged cerebellar imaging data. *PLOS ONE*, *10*(7), e0133402. https://doi.org/10.1371/journal.pone.0133402

Guell, X., Arnold Anteraper, S., Gardner, A. J., Whitfield-Gabrieli, S., Kay-Lambkin, F., Iverson, G. L., Gabrieli, J., & Stanwell, P. (2020). Functional connectivity changes in retired rugby league players: A data-driven functional magnetic resonance imaging study. *Journal of Neurotrauma*, neu.2019.6782. https://doi.org/10.1089/neu.2019.6782

Guell, X., Schmahmann, J. D., Gabrieli, J. D., & Ghosh, S. S. (2018). Functional gradients of the cerebellum. *ELife*, *7*, e36652. https://doi.org/10.7554/eLife.36652

Henson, R. N., Campbell, K. L., Davis, S. W., Taylor, J. R., Emery, T., Erzinclioglu, S., & Kievit, R. A. (2016). Multiple determinants of lifespan memory differences. *Scientific Reports*, *6*(1), 32527. https://doi.org/10.1038/srep32527

Katsumi, Y., Kondo, N., Dolcos, S., Dolcos, F., & Tsukiura, T. (2021). Intrinsic functional network contributions to the relationship between trait empathy and subjective happiness. *NeuroImage*, *227*, 117650. https://doi.org/10.1016/j.neuroimage.2020.117650

Kawagoe, T., Onoda, K., & Yamaguchi, S. (2019). Subjective memory complaints are associated with altered resting-state functional connectivity but not structural atrophy. *NeuroImage: Clinical*, *21*, 101675. https://doi.org/10.1016/j.nicl.2019.101675

Morris, T. P., Chaddock-Heyman, L., Ai, M., Anteraper, S. A., Castañon, A. N., Whitfield-Gabrieli, S., Hillman, C. H., McAuley, E., & Kramer, A. F. (2021). Enriching activities during childhood are associated with variations in functional connectivity patterns later in life. *Neurobiology of Aging*, *104*, 92–101. https://doi.org/10.1016/j.neurobiolaging.2021.04.002

Takamiya, A., Kishimoto, T., Hirano, J., Nishikata, S., Sawada, K., Kurokawa, S., Yamagata, B., Kikuchi, T., & Mimura, M. (2021). Neuronal network mechanisms associated with depressive symptom improvement following electroconvulsive therapy. *Psychological Medicine*, *51*(16), 2856–2863. https://doi.org/10.1017/S0033291720001518

Whitfield-Gabrieli, S., Ghosh, S. S., Nieto-Castanon, A., Saygin, Z., Doehrmann, O., Chai, X. J., Reynolds, G. O., Hofmann, S. G., Pollack, M. H., & Gabrieli, J. D. E. (2016). Brain connectomics predict response to treatment in social anxiety disorder. *Molecular Psychiatry*, *21*(5), 680–685. https://doi.org/10.1038/mp.2015.109
